# Outpatient treatment of pneumonia in a setting with and without an infectious disease doctor

**DOI:** 10.3325/cmj.2023.64.45

**Published:** 2023-02

**Authors:** Marija Kusulja, Maša Žarković, Nikola Kudoić, Monika Mudrovčić, Natalija Sovina Stražičić, Ivan Gornik, Vladimir Krajinović

**Affiliations:** 1 Dr Fran Mihaljević University Hospital for Infectious Diseases, Zagreb, Croatia; 2 Institute of Social and Preventive Medicine, University of Bern, Bern, Switzerland; 3 Zagreb University Hospital Center, Zagreb, Croatia; 4 Zagreb University School of Medicine, Zagreb, Croatia

## Abstract

**Aim:**

To compare the outpatient treatment of community acquired pneumonia (CAP) by infectious disease doctors (IDDs) and doctors of other specialties (nIDDs).

**Methods:**

We retrospectively identified 600 outpatients with CAP: 300 treated by IDDs and 300 by nIDDs in two tertiary hospitals during 2019. The two groups were compared in terms of adherence to guidelines, antibiotic group prescription, frequency of combined treatment, and treatment duration.

**Results:**

IDDs prescribed significantly more first-line treatment (*P* < 0.001) and alternative treatment (*P* = 0.008). NIDDs prescribed more reasonable (*P* < 0.001) and unnecessary (*P* = 0.002) second-line treatment, and inadequate treatment (*P* = 0.004). IDDs significantly more frequently prescribed amoxicillin (*P* < 0.001) for typical and doxycycline (*P* = 0.045) for atypical CAP, while nIDDs significantly more frequently prescribed amoxicillin-clavulanate (*P* < 0.001) for typical and fluoroquinolones for both typical (*P* < 0.001) and atypical (*P* < 0.001) CAP. No significant differences were found in the frequency of combined treatment, which exceeded 50% in both groups, or in treatment duration.

**Conclusions:**

Outpatient treatment of CAP in the absence of IDDs meant more broad-spectrum antibiotic prescription and more disregard for national guidelines. Our results highlight the need for antibiotic stewardship, especially in settings with no IDDs.

Community-acquired pneumonia (CAP) is one of the most common diagnoses in patients discharged from the emergency department (1). These patients are seen by doctors with a wide range of medical specialties. Treatment of CAP is usually empirical, based on knowledge of the most common causative pathogens and their antimicrobial susceptibility, with no routine etiological diagnostic tests done. Guidelines on CAP treatment (especially outpatient treatment) vary widely across the world, with options including penicillins, cephalosporins, macrolides, doxycycline, and fluoroquinolones (2-7).

The most recent Croatian national guidelines for the treatment of CAP were published in 2017 (8). They distinguish between typical bacterial pneumonia and atypical pneumonia based on clinical presentation and suggest different treatments for each. The recommended first-line outpatient treatment for typical bacterial pneumonia is amoxicillin for a duration of 7 to 10 days, with alternative choices including amoxicillin-clavulanate, second and third generation of cephalosporins, or respiratory fluoroquinolones. Amoxicillin-clavulanate is preferred over amoxicillin in patients with risk factors for causative agents other than *S. pneumoniae*, patients older than 65 years, patients from nursing homes, patients hospitalized within the previous 90 days, and those with chronic lung diseases. The recommended treatment for atypical pneumonia is azithromycin for a duration of 3 days, with the alternatives including clarithromycin or doxycycline. The guidelines advise against combined therapy (use of two antibiotics to cover both typical and atypical pneumonia) in cases with no clear clinical distinction in outpatients. Instead, an initial amoxicillin monotherapy is recommended with close follow-up and possible addition of a second antibiotic in case of treatment failure.

Given that Croatia has hospitals with exclusively infectious disease doctors (IDDs) and those with solely non-infectious disease doctors (nIDDs), we aimed to examine adherence to guidelines and compare the choice of antimicrobial agents that IDDs and nIDDs use to treat outpatient CAP.

## Patients and methods

We retrospectively searched the emergency department visit databases of Dr Fran Mihaljević University Hospital for Infectious Diseases (UHID) and University Hospital Center Zagreb (UHCZ) to identify adult patients examined in the emergency department and discharged home with a primary diagnosis of pneumonia and oral treatment only from January 1 to December 31, 2019. Pneumonia was diagnosed as typical or atypical at the discretion of the attending physician. The participants were from the UHID, where only IDDs attend to patients, and from UHCZ, which employs no IDDs so that their patients are attended to by internal medicine and emergency medicine doctors. We did not include patients who were hospitalized and those treated with intravenous antibiotics in the day hospital. Based on the total number of patients treated for pneumonia as outpatients in both centers (1502 in UHID and 1645 in UHCZ) in 2019 and allowing a 95% confidence interval and a 5% margin of error, the needed sample size was 300 patients from each center. Stratified randomization by month was used to recruit patients equally across the year. From medical records, we obtained data on patients’ demographics, the attending doctor’s specialty, comorbidities, allergies to antibiotics, the antibiotic prescribed, the duration of treatment, and the readmission to either hospital’s emergency department. We compared the prescribed treatment with the national guidelines, referring to the treatment of mild pneumonia (which we assumed all these cases to be, as per the physician’s decision to treat via the outpatient route). We categorized the treatment as either “first-line;” “reasonable second-line” if patients reported an allergy to the first-line medication or if they reported risk factors justifying an amoxicillin-clavulanate prescription; “unnecessary second-line” if no obvious reason for deviating from the first line was found; “alternative” if the treatment option was not mentioned in the guidelines but was expected to be effective based on the known susceptibility of the most common causative pathogens (9); and “inadequate” if the treatment was not mentioned in the guidelines and was not expected to be effective. We also compared the choices of treatment between the IDDs and nIDDs in terms of the antibiotic groups and the prescribed length of treatment. Where dual treatment was prescribed, the single most appropriate antibiotic prescribed was taken into account in the analysis of treatment concordance with the guidelines and antibiotic group comparisons between prescribing physicians. This was possible since in all the dually treated cases of typical pneumonia, the second antibiotic was azithromycin, and in the single case of dually treated atypical pneumonia it was a cephalosporin. This study was approved by the Ethics Committees of both UHID and UHCZ.

### Statistical analysis

The normality of distribution was assessed with a Shapiro-Wilk test and a visual inspection. Ordinal and interval variables are presented as medians and interquartile ranges. The significance of differences in ordinal and interval data was assessed with a Mann-Whitney test. Hierarchical binomial logistic regression was used to assess whether the prescription rate of amoxicillin-clavulanate remained significantly different between IDDs and nIDDs after controlling for the patient's risk factors for causative agents of typical pneumonia other than the most common *S. pneumoniae*. The models fit the data (Hosmer and Lemeshow test, *P* > 0.05). No outliers were found. When performing χ^2^ tests on tables larger than 2 × 2, adjusted standardized residuals were calculated to determine which specific cells significantly deviated from the expected frequencies. The alpha level was set at 5%. The statistical analysis was performed with SPSS, version 26.0 (IBM Corp., Armonk, NY, USA).

## Results

The study enrolled 600 patients (50.5% female): 300 seen by IDDs and 300 by nIDDs. The median age of patients was 53 years (IQR 32.25, 71). Overall, 150 patients were diagnosed with atypical pneumonia (136 by IDDs and 14 by nIDDs), while 450 were either explicitly stated or assumed to have typical bacterial pneumonia (164 diagnosed by IDDs and 286 by nIDDs) (Table 1). A total of 189 (31.5%) patients received first-line therapy, 388 (64.7%) second-line therapy – of which 147 (37.9%) reasonably and 241 (62.1%) unnecessarily so. Nine patients (1.5%) received alternative and 14 (2.3%) received inadequate treatment.

**Table 1:** Demographic data and comorbidities

	Infectious disease doctors		Pneumonia		All cases (n = 600)
	no (n = 300)	yes (n = 300)	atypical (n = 150)	typical (n = 450)	
Sex (female), n (%)	156 (52.0)	147 (49.0)	77 (51.3)	226 (50.2)	303 (50.5)
Age, median (interquartile range)	70 (50, 80)	41 (30, 54.75)	40 (28, 51.5)	61 (38, 76)	53 (32.25, 71)
Nursing home residents, n (%)	20 (6.7)	2 (0.7)	1 (0.7)	21 (4.7)	22 (3.7)
Recent hospitalization (90 days), n (%)	32 (10.7)	3 (1.0)	0 (0.0)	35 (7.8)	35 (5.8)
Chronic lung disease, n (%)	37 (12.3)	10 (3.3)	6 (4)	41 (9.1)	47 (7.8)

IDDs and nIDDs differed significantly in terms of adherence to guidelines, ie, the frequency of different therapy-line prescriptions (χ^2^ (4) = 155.55, *P* < 0.001) (Table 2). The effect was large (V = 0.588). IDDs more frequently prescribed first-line (*P* < 0.001) and alternative therapy (*P* = 0.008), while nIDDs more frequently prescribed second-line treatment, both when it was reasonable (*P* < 0.001) and when it was unnecessary (*P* = 0.002). NIDDs also significantly more frequently prescribed inadequate treatment (*P* = 0.004). The main point of differentiation of second-line therapy being reasonable or unnecessary was the patient’s history of drug allergies. In our data set, IDDs were less likely than nIDDs to describe the allergic reaction to any antibiotic as “unknown” (χ^2^ (1) = 9.90, *P* = 0.002; V = 0.384).

**Table 2:** Adherence to national guidelines for treatment of community-acquired pneumonia in outpatients*

			Infectious disease doctors		
Diagnosis	Line of treatment		yes	no	χ^2^ (df), V, p
Typical pneumonia	first line	N (%)	72 (43.9)	3 (1.0%)	155.55, (4), 0.588, <0.001
		*P*	<0.0010		
	reasonable second line		27 (16.5)	120 (42.0)	
		*P*	<0.001		
	unnecessary second line	N (%)	61 (37.2)	149 (52.1)	
		*P*	0.0020		
	alternative (effective)	N (%)	4 (2.4)	0 (0.0)	
		*P*	0.008		
	inadequate		0 (0.0)	14 (4.9)	
		*P*	0.004		
Atypical pneumonia	first line	N (%)	72 (43.9)	3 (1.0)	18.37, (2), 0.350, 0.001
		*P*	0.813		
	reasonable second line	N (%)	0	0	
	unnecessary second line	N (%)	31 (22.8)	0 (0.0)	
		*P*	0 0.045		
	alternative (effective)	N (%)	2 (1.5)	3 (21.4)	
		*P*	0 <0.001		
	inadequate	N (%)		0	

*Abbreviations: df – degrees of freedom, V – Cramer's V effect size.

For typical pneumonia, IDDs and nIDDs significantly differed in terms of antibiotic group choices (χ2 (3) = 158.70, *P* < 0.001), with a large effect size (V = 0.599) (Table 3). IDDs were more likely to prescribe amoxicillin (*P* < 0.001), while nIDDs were more likely to prescribe amoxicillin-clavulanate (*P* < 0.001) and fluoroquinolones (*P* < 0.001). Differences in the prescription rates of cephalosporins were not significant (*P* = 0.188). While nIDDs prescribed amoxicillin-clavulanate more often than IDDs, the effect can be attributed to the fact that nIDDs more often treated patients with risk factors for less common causative agents. This was supported by the fact that both the prescription of amoxicillin-clavulanate (χ2 (1) = 32.87; *P* < 0.001) and risk factors (χ2 (1) = 84.63; *P* < 0.001) independently significantly predicted the physician’s specialty. In addition, the model created including risk factors for CAP caused by less common causative agents as a control variable and the prescription of amoxicillin-clavulanate as a second step of a predictor model was significant (χ2 (1) = 84.69; *P* < 0.001). However, the risk factors remained a significant predictor (odds ratio [OR] 7.1, confidence interval [CI] 95% 4.0-13.9, *P* < 0.001), but the added predictor of the prescription of amoxicillin-clavulanate was not significant and did not result in a better prediction (OR 0.9, CI 95% 0.4-1.9, *P* = 0.800). The explained variance was almost the same as in the model containing only the risk factors (R2 Nagelkerke = 0.235).

**Table 3:** Prescribed treatment by antibiotic group*

			Infectious disease doctors		
Diagnosis	Antibiotic group		yes	no	χ^2^ (df), V, *P*
Typical pneumonia	amoxicillin	N (%)	72 (16.3)	3 (0.7)	158.70, (3), 0.599, <0.001
		*P*	<.001		
	co-amoxiclav	N (%)	66 (14.9)	180 (40.6)	
		*P*	<.001		
	cephalosporins	N (%)	15 (3.4)	17 (3.8)	
		*P*	0.188	0.188	
	fluoroquinolones	N (%)	7 (1.6)	83 (18.7)	
		*P*	<.001		
Atypical pneumonia	fluoroquinolones	N (%)	2 (1.5)	3 (21.4)	18.37 (2), 0.350, 0.001
		*P*	<0.001		
	macrolides	N (%)	103 (75.7)	11 (78.6)	
		*P*	0.813		
	doxycycline	N (%)	31 (22.8)	0 (0.0)	
		*P*	0.045		

*Abbreviations: df – degrees of freedom, V – Cramer's V effect size.

For atypical pneumonia, IDDs and nIDDs significantly differed in terms of antibiotic group choices as well (χ2 (2) = 18.37, *P* = 0.001), with a medium effect size (V = 0.350) (Table 3). Standardized adjusted residuals revealed that IDDs were more likely to prescribe doxycycline (*P* = 0.045), while nIDDs were more likely to prescribe fluoroquinolones (*P* < 0.001). The prescription rates of macrolides did not significantly differ (*P* = 0.813).

Three hundred patients (50%) received dual antibiotic treatment, 63% (n = 189) of whom were treated by nIDDs and 37.3% (n = 112) by IDDs. Only one patient with atypical and 66.9% (n = 301) of patients with typical pneumonia were prescribed two antibiotics. Although dual treatment was more commonly prescribed by nIDDs than by IDDs in absolute terms, when considering the difference in the number of typical pneumonias treated by each group (68.3% of all typical pneumonias treated by IDD received two antibiotics vs 66.1% of all typical pneumonias treated by nIDDs), no significant differences in prescribing dual therapy were found (χ2 (1) = 0.12, *P* = 0.756). The most common second antibiotic prescribed was azithromycin.

IDDs or nIDDs did not significantly differ in terms of the duration of therapy they prescribed, both for typical (χ2 (1) = 0.03, *P* = 1) and atypical (χ2 (1) = 1.61, *P* = 0.361) pneumonia. They also did not differ in how much the duration of their prescription deviated from the guidelines, both when the guidelines proposed 7-10 (U = 18579.5, *P* = 0.629) and 3 days of therapy (U = 647.0, *P* = 0.780).

They also did not differ in the occurrence rates of readmission to the emergency department due to clinical deterioration, both for cases of typical (χ2 (1) = 1.33, *P* = 0.327) and atypical pneumonia (χ2 (1) = 0.21, *P* = 1). There were also no significant differences in the number of days from the beginning of therapy to an emergency department revisit, if the revisit occurred (U = 123.0, *P* = 0.635).

## Discussion

In our study, IDDs adhered to national guidelines more, but also used more alternative treatments (effective, but not mentioned in the guidelines). They were more likely to prescribe amoxicillin for typical and doxycycline for atypical pneumonia. NIDDs less commonly adhered to guidelines, and more frequently prescribed second-line treatment and inadequate treatment options. They prescribed more amoxicillin-clavulanate for typical bacterial pneumonia, and more fluoroquinolones for both types of pneumonia.

The empirical use of fluoroquinolones has been identified in recent years as exceedingly problematic. The reasons are manifold – from rare but concerning side effects (10,11), a role in the pathogenesis of the *Clostridioides difficile* epidemic (12) to their effect in selecting bacteria resistant to both fluoroquinolones and beta-lactam antibiotics (13). The overuse of broad-spectrum antibiotics, in this case especially fluoroquinolones, may be thwarted by antibiotic stewardship, which has been shown to reduce inappropriate antibiotic use even with a non-restrictive approach (14,15).

The fact that nIDDs in our study more often prescribed second-line antibiotics may be explained partly by the fact that nIDDs saw more patients with comorbidities warranting the prescription of amoxicillin-clavulanate. However, nIDDs described patient allergies as “unknown” more frequently. This led them to prescribe a reasonable second-line treatment option, but it may have been avoided if a more detailed patient history had been taken. Another reason for nIDD prescribing more second-line and broad-spectrum antibiotics might be that nIDD are less often able to follow-up patients seen in their emergency department.

Our results agree with previously published studies showing that the presence of IDDs (either as the main or consulting physician) was associated with more rational treatment, ie, treatment in accordance with antibiotic stewardship principles, in patients with various infections hospitalized in and outside of intensive care units (16-19).

In our study, dual antibiotic treatment was prescribed in 50% of all cases and in 66.9% of typical bacterial pneumonias, with no significant difference in the prescription rates between IDDs and nIDDs. This is against the recommendations for the treatment of mild pneumonia in the national guidelines, and it is only recommended in severe pneumonia or after a lack of clinical improvement in mild pneumonia treated by monotherapy. The level of diagnostic certainty was found to be positively related to guideline adherence in CAP treatment (20). The high prevalence of dual therapy in our study may be explained by difficulties in differentiation between typical and atypical pneumonia based on a single outpatient emergency department visit.

A small number of patients in our study returned to either hospital due to clinical deterioration. This suggests that any differences in treatment choices between IDDs and nIDDs did not affect the recovery from mild pneumonia. However, inappropriate use of antibiotics in uncomplicated acute respiratory infections has been shown to lead to a selection of resistant intestinal *Enterobacteriaceae *(21).

The advantages of our study include a cohort with CAP treated as outpatients with oral antibiotics only, as well as a selection of hospitals with and without IDDs. This allowed us to examine the differences in the prescription of antibiotics for a very common infection in settings with and without IDDs.

The limitations of our study are a relatively small sample and the participation of only two centers. In our statistical analysis, no *P* value adjustments for multiple synchronous comparisons were done. We also noted a large difference in the number of pneumonias classified (and treated) as atypical by IDDs vs nIDDs. Our sample was random, suggesting that IDDs simply diagnosed more atypical pneumonias. This is perhaps due to the fact that IDDs saw younger patients with less comorbidities, who are more prone to atypical pneumonia. However, it may also be argued that IDDs were more likely to distinguish between typical and atypical CAP, and were more prone to follow guidelines for treatment which differentiate the two clinical entities.

In conclusion, our study showed that nIDDs prescribed more broad-spectrum antibiotics to outpatients with CAP, while IDDs were more inclined to use first-line treatment according to the national guidelines. Both groups opted for dual treatment in more than a half of the patients with typical pneumonia, against the recommendations in national guidelines. Although the inappropriate use of antibiotics did not result in clinical deterioration in individual patients in this study, there is a concern that this may fuel the growing threat of antibiotic resistance. Antibiotic stewardship is needed to improve the practice of irrational use of antibiotics, especially in settings with no IDDs.
